# Comparative lipidomics of iPSC-derived microglia protocols reveal lipid droplet and immune differences mediated by media composition

**DOI:** 10.1016/j.stemcr.2025.102779

**Published:** 2026-01-08

**Authors:** Aiko Toda Robert, Amanda McQuade, Sascha J. Koppes-den Hertog, Lena Erlebach, Deborah Kronenberg-Versteeg, Martin Kampmann, Martin Giera, Rik van der Kant

**Affiliations:** 1 Department of Functional Genomics, Center for Neurogenomics and Cognitive Research (CNCR), VU Amsterdam, Amsterdam, the Netherlands; 2 Alzheimer Center Amsterdam, Department of Neurology, Amsterdam Neuroscience, Amsterdam University Medical Center, Amsterdam, the Netherlands; 3 Institute for Neurodegenerative Diseases, University of California, San Francisco, San Francisco, CA, USA; 4 German Center for Neurodegenerative Diseases (DZNE), Tübingen, Tübingen, Germany; 5 Department of Cellular Neurology, Hertie Institute for Clinical Brain Research, University of Tübingen, Tübingen, Germany; 6 Graduate Training Center of Neuroscience, School of Cellular and Molecular Neuroscience, Eberhard Karls University Tübingen, Tübingen, Germany; 7 Department of Biochemistry and Biophysics, University of California, San Francisco, San Francisco, CA, USA; 8 Center for Proteomics and Metabolomics, Leiden University Medical Center, Leiden, the Netherlands

**Keywords:** microglia, iPSC, lipidomics, lipid droplet, lipid metabolism, triglycerides, neuroinflammation

## Abstract

Altered microglial lipid metabolism is heavily implicated in Alzheimer’s disease (AD) and aging. Recently, protocols were developed to generate human induced pluripotent stem cell-derived microglia-like cells (iMGL) to study microglial function *in vitro,* including embryoid body-based methods and induced transcription factor (iTF)-dependent approaches. Here, we performed comparative lipidomics on iMGL from these methods and report major differences in multiple lipid classes, including triglycerides (TGs), a storage form of fatty acids implicated in microglial reactivity. TGs are strongly increased in iTF microglia due to the absence of a media supplement (B-27). Supplementing iTF microglia with B-27, or its component L-carnitine, reduces TGs and promotes a homeostatic state. B-27 also renders iTF microglia metabolically responsive to immune stimuli. Overall, our data show that iMGL differentiation methods have a major impact on microglial lipidomes and warrant attention when studying AD and neuroinflammatory processes involving lipids.

## Highlights

• iMGL from different generation methods shows major differences in lipid metabolism
• B-27 supplement drastically alters the iMGL lipidome and microglial state markers
• The B-27 effect is mainly driven by L-carnitine, a cofactor in mitochondrial fatty acid import

## Teaser

Van der Kant and colleagues uncover that the lipidome of iPSC-derived microglia is strongly affected by the selection of differentiation protocol and media supplements. The authors show that the B-27 supplement, and specifically the L-carnitine present in B-27, reduces fatty acid storage in triglycerides, thereby decreasing lipid droplet content and inducing a more homeostatic microglial state.

## Introduction

One of the pathological hallmarks of Alzheimer’s disease (AD) includes microgliosis. Microgliosis describes the process by which microglia respond to a wide range of cues in the central nervous system (CNS) by adopting a plethora of transcriptional profiles, often accompanied by alterations in cytokine and chemokine secretion (Heneka et al., 2015; Leng and Edison, 2021). Genome-wide association studies have identified over 75 loci that implicate microglia and altered lipid metabolism in AD pathogenesis (Bellenguez et al., 2022; Kunkle et al., 2019; Lambert et al., 2013; Sims et al., 2017). For example, AD risk genes *TREM2* and *PLCγ2* are highly expressed in microglia and key regulators of brain lipid metabolism (Andreone et al., 2020; Sims et al., 2017; Tsai et al., 2022; 2023). Alterations in lipid metabolism have been correlated to various microglial states in the diseased and aging brain. For instance, the major sporadic AD risk gene *APOE*, a central player in lipoprotein-mediated lipid transport, is normally highly expressed in astrocytes but becomes strikingly upregulated in microglia during AD progression in so-called disease-associated microglia (DAM) (Keren-Shaul et al., 2017; Krasemann et al., 2017). DAM locate in proximity to amyloid-β (Aβ) plaques and exhibit increased expression of genes regulating lipid metabolism, including *ApoE*, *Trem2* and *Lpl*, which encodes for an enzyme involved in triglyceride (TG) metabolism (Keren-Shaul et al., 2017). In aged wild-type mice and in 5xFAD mice, lipid droplet-accumulating microglia (LDAM) containing high levels of TGs, the storage form of excess fatty acids (FAs), have been observed (Marschallinger et al., 2020; Prakash et al., 2025). LDAM were also identified in human *postmortem* AD brains and in induced pluripotent stem cell (iPSC)-derived microglia like cells (iMGL) exposed to Aβ fibrils, tau pathology, as well as in chimeric 5xFAD mice bearing human microglia (Claes et al., 2021; Haney et al., 2024; Li et al., 2024; Prakash et al., 2025).

Owing to the inaccessibility of primary human microglia from living patients, the past decade has seen a surge in protocols to generate iMGL (Sabogal-Guáqueta et al., 2020; Speicher et al., 2019). We recently showed that iPSC-derived brain cell types, including iMGL, have distinct lipidomes that partially reflect those of mouse brain-derived cells (Feringa et al., 2025). Most current protocols to generate iMGL mimic microglial ontogeny either via the formation of embryoid bodies (EBs), which subsequently yield yolk sac-derived myeloid progenitors, or via the generation of hematopoietic progenitors. After the addition of growth factors, the resulting iMGL (hereafter termed EB microglia) share some key characteristics with primary fetal and adult human microglia including the expression of microglial signature genes, cytokine, and chemokine release upon pro-inflammatory stimuli, phagocytosis of synaptosomes or fibrillar Aβ and migration toward injury sites (Abud et al., 2017; Brownjohn et al., 2018; Douvaras et al., 2017; Garcia-Reitboeck et al., 2018; Haenseler et al., 2017; McQuade et al., 2018; Muffat et al., 2016; Pandya et al., 2017; Takata et al., 2017).

However, this EB-based method is rather lengthy, costly, and variation in yield per differentiation reduces its scalability. Recently, a protocol for a rapid and simplified generation of iMGL (hereafter iTF microglia) was developed. This method relies on the doxycycline (dox)-inducible expression of microglial fate-determining transcription factors MAFB, PU.1, CEBPα, CEBPβ, IRF5, and IRF8 and allows for the generation of iMGL in 8 days in a highly scalable manner, while preserving key microglial features for *in vitro* modeling (Dräger et al., 2022).

As lipid metabolism is important for microglial function, in the present study, we compared the lipidomes of EB and iTF microglia. We find that iTF microglia have a high lipid droplet (LD) load with a strong enrichment in TGs and its precursors compared to EB microglia. We show that differences in TG levels between protocols can largely be attributed to the microglia maturation media composition: specifically, L-carnitine (L-car) found in B-27 supplement is associated with lower TG levels. Overall, our experiments show that methods and supplements adopted to generate iMGL have a major effect on the resulting microglial lipidome and warrant consideration when modeling microglial function in AD and other neurodegenerative diseases.

## Results

### Properties of embryoid body- and induced transcription factor-dependent differentiation protocols

**Figure 1:**
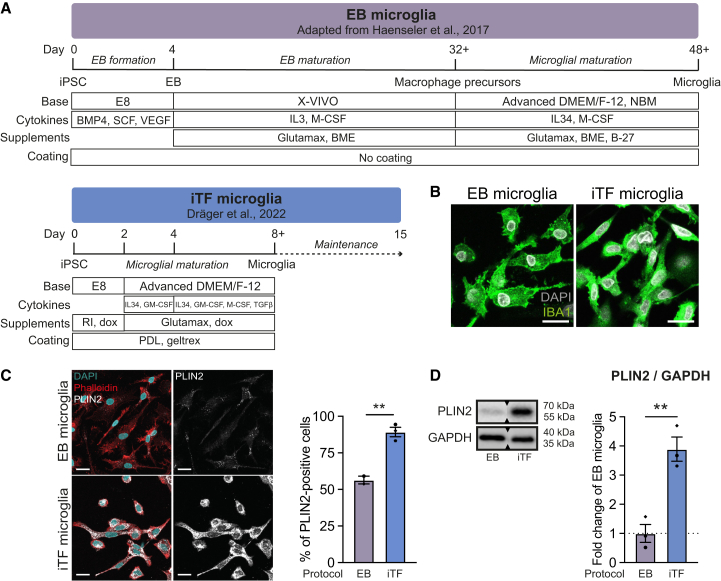
Comparison of iPSC-derived EB and iTF microglia (WTC11) (A) Overview of the differentiation process for EB and iTF microglia. (B) Representative confocal microscopy images of EB and iTF microglia stained for IBA1. Scale bars = 25 μm. (C) Representative confocal images of LDs (PLIN2) in EB and iTF microglia stained with phalloidin (cell outline) and DAPI (nuclei). Quantification of PLIN2-positive cells. Unpaired *t* test. Scale bars = 25 μm. *N* = 2 (EB) and *N* = 3 (iTF) independent cultures, with >500 cells imaged per well. (D) Representative Western blot (WB) and quantification of PLIN2 levels. Paired *t* test. *N* = 3 independent cultures. *Sections where the blot was cut are indicated with triangles.* (C and D) Data shown as mean ± SEM. *Symbols denote independent cultures.∗∗p<0.005.* See also Figure S1.

Given the different approaches taken to generate EB and iTF microglia, we set out to characterize and compare the protocols (Figure 1A). Microglia generated with both methods express the canonical microglial marker protein IBA1 (Figure 1B). Next, we evaluated the expression of microglia and macrophage marker genes by quantitative reverse transcription polymerase chain reaction (qRT-PCR) (Figure S1A). While myeloid markers *CSF1R* and *CX3CR1*, and microglia marker *P2RY12* were highly expressed in both EB and iTF microglia, the macrophage-specific marker *LYVE1* exhibited low expression in iTF microglia but higher expression in EB microglia (Figure S1A). Microglia marker *TMEM119* mRNA was higher in iTF microglia, as was the expression of genes involved in interferon signaling (Figure S1B). Strikingly, iTF microglia had more LDs and higher expression of perilipin-2 (PLIN2), a protein enriched in LD membranes (Figures 1C and 1D). In conclusion, we show that iMGL generated using both EB and iTF protocols exhibit key microglial signatures in monoculture but differ substantially in their LD load.

### Lipidomic profiling uncovers elevated neutral lipids in induced transcription factor microglia

**Figure 2:**
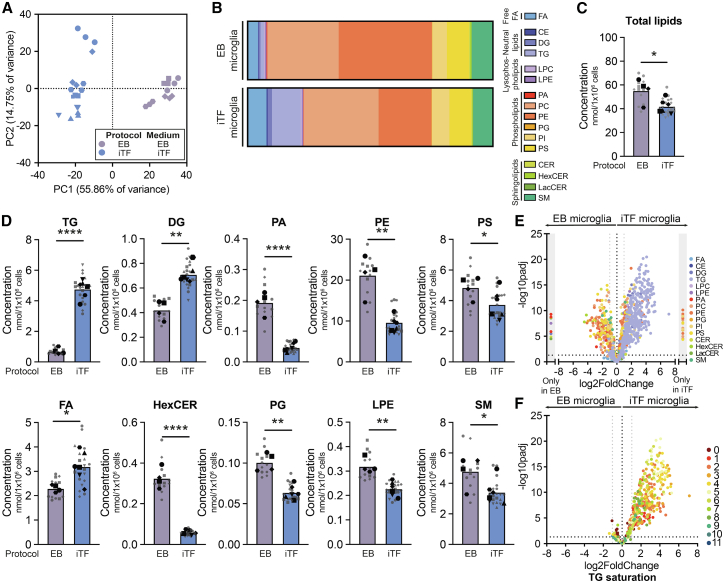
Lipidomic analysis of iPSC-derived EB and iTF microglia (WTC11) (A) PCA plot of unbiased lipidomics. (B) Average distribution of lipid classes as a percentage of total lipids. (C) Total lipid concentration in nmol per 1 × 10^6^ cells. Unpaired *t* test. (D) Lipid class concentration in nmol per 1 × 10^6^ cells. Unpaired t-tests with FDR (Benjamini-Hochberg) correction for multiple comparisons. (E) Volcano plot shows log2 fold change of altered lipid species. (F) Volcano plot shows log2 fold change of altered TG species. Color coding represents the number of double bonds. (A–F) *N* = 4 and *N* = 6 independent cultures for EB and iTF microglia, respectively, with 3 technical replicates each. (C and D) *Symbols denote independent cultures. Technical replicates are in gray. The mean of technical replicates is in black.* ∗*p* < 0.05, ∗∗*p* < 0.005, ∗∗∗∗*p* < 0.0001. LPC = lysophosphatidylcholine, LPE = lysophosphatidylethanolamine, PG = phosphatidylglycerol, PI = phosphatidylinositol, CER = ceramide, LacCER = lactosylceramide, SM = sphingomyelin. See also Figure S2.

Next, we performed lipidomic analysis on EB and iTF microglia from two independent iPSC lines (WTC11 and KOLF2.1J) (Figures 2A–2F and S2A–S2E). We detected 1009 common lipid species across all samples, spanning 16 lipid classes. Total lipid concentration was higher in EB microglia (Figure 2C). After normalizing to total lipid content, EB and iTF microglia clearly separated by principal component analysis (PCA) (Figures 2A and S2C) and at the lipid class level (Figures 2B and S2D). Phospholipids, particularly phosphatidylcholine (PC) and phosphatidylethanolamine (PE), were most abundant in both EB and iTF microglia, constituting ∼82% and ∼68% of their lipidome, respectively. Although TGs only made up ∼1% of the lipidome in EB microglia, over 11% of the iTF microglial lipidome consisted of TGs, which was also reflected in higher absolute TG concentrations in iTF microglia (Figures 2B, 2D, and S2B). Similarly, TG precursors diglycerides (DGs) were more abundant in iTF microglia (Figure 2D). In contrast, EB microglia contained higher concentrations of phosphatidic acid (PA), PE, and phosphatidylserine (PS) (Figures 2D, S2D, and S2E). We also noted higher levels of hexosylceramides (HexCERs) in EB microglia (Figures 2D and S2E). Other differences in lipid classes appeared line- rather than protocol-specific (Figures 2D, S2A, S2B, and S2E).

After analyzing changes at the lipid class level, we evaluated differences between protocols at the level of individual lipid species (Figure 2E). We observed that most species within the TG, DG, PA, and HexCER groups differed between protocols, indicating a general shift in lipid class rather than specific lipid species. Indeed, most TG species measured were increased in the iTF microglia, without the preferential accumulation of species with a specific saturation level (Figure 2F). Taken together, our comparative analysis shows that iTF microglia have considerably higher levels of lipids stored in LDs.

### Microglia maturation medium, rather than the induction method, affects triglyceride levels in induced pluripotent stem cell-derived microglia like cells

**Figure 3:**
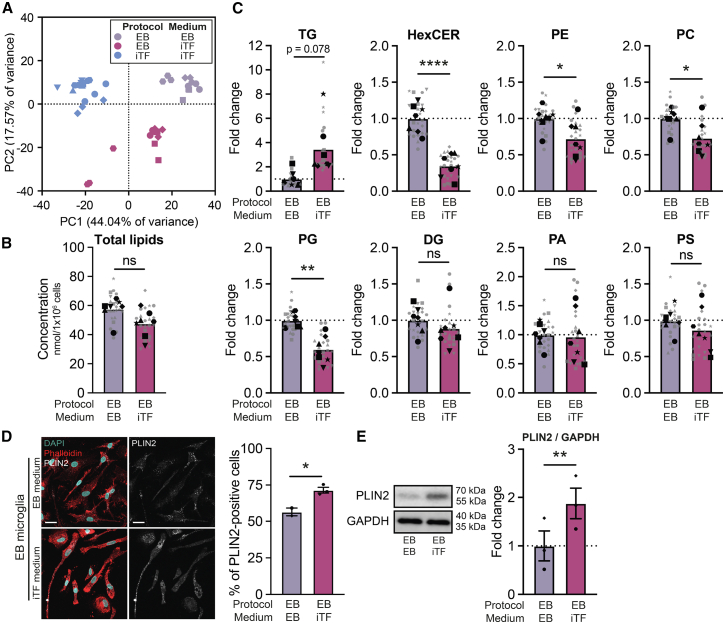
iTF medium increases the TG content of EB microglia (WTC11) (A) Representative PCA plot of unbiased lipidomics from one lipidomic run. (B) Total lipid concentration in nmol per 1 × 10^6^ cells. Paired *t* test. (C) Lipid class concentration represented as fold change of EB microglia in EB medium (EB-EB). Paired t-tests with FDR (Benjamini-Hochberg) correction for multiple comparisons. (D) Representative confocal microscopy images of LDs (PLIN2) in EB-EB and EB-iTF microglia stained with phalloidin (cell outline) and DAPI (nuclei). Quantification of PLIN2-positive cells. Unpaired *t* test. Scale bars = 25 μm. *N* = 2 (EB-EB) and *N* = 3 (EB-iTF) independent cultures, with >500 cells imaged per well. (E) Representative WB and quantification of PLIN2 levels. Paired *t* test. *N* = 3 independent cultures. (D and E) Data shown as mean ± SEM. *Symbols denote independent cultures.* (B and C) *N* = 7 independent cultures with 3 technical replicates each. *Symbols denote independent cultures. Technical replicates are in gray. The mean of technical replicates is in black.ns = non-significant, ∗p<0.05, ∗∗p<0.005, ∗∗∗∗p<0.0001.The EB-EB data in* (A–C) *are the same (with additional replicates) as seen in*Figures 2A–2F*. The microscopy and WB images (and corresponding quantifications) for the EB-EB condition in* (D and E) *are the same as seen in*Figures 1C and 1D. (See also Figure S3).

To determine why iTF microglia have higher TGs, we performed lipidomic analysis of iMGL generated using the EB protocol matured in iTF medium (EB-iTF microglia) (Figures 3A–3C and S3). PCA clearly distinguished the three groups, where EB-iTF microglia formed an intermediate cluster (Figure 3A). While culturing EB microglia in iTF medium did not affect the overall lipid concentration (Figure 3B), we observed an increase in TGs, indicating that the medium drives the accumulation of these lipids (Figure 3C). Accordingly, the proportion of PLIN2-positive cells and PLIN2 protein levels were also increased in EB-iTF microglia (Figures 3D and 3E). Interestingly, we noted a decrease in HexCERs with the medium switch (Figure 3C).

TGs are a storage form of excess FAs, and exogenous FAs are often added to culture media. Therefore, we hypothesized that differences in culture media lipid composition might drive the increased TG storage in iTF microglia. To examine this, we compared the lipid composition of EB and iTF maturation media (Figure S4A). FAs were the most abundant lipids supplemented in both EB (11691 pmol/mL) and iTF media (9508 pmol/mL) (Figures S4B and S4C). EB medium also contained higher final concentrations of TGs and DGs (Figure S4C). These observations argue against the TG increase in iTF microglia directly reflecting the medium’s lipid sources (Figure S4D). Overall, these data indicate that the maturation medium, rather than the induction method, has a major effect on the TG content of the resulting iMGL.

### The medium supplement B-27 regulates triglyceride accumulation

**Figure 4:**
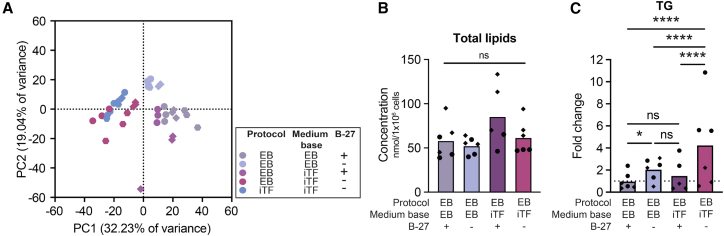
Omitting B-27 supplement increases TG levels (WTC11 and KOLF2.1J) (A) Representative PCA plot of unbiased lipidomics from one lipidomic run (WTC11). *Symbols denote independent cultures with 3 technical replicates each*. (B) Total lipid concentration in nmol per 1 × 10^6^ cells. One-way ANOVA, Tukey’s multiple comparisons post-hoc test. (C) Lipid class concentration represented as fold change of EB-EB microglia. Two-way ANOVA, Tukey’s multiple comparisons post-hoc test. (B and C) *N* = 3 independent cultures from WTC11 and KOLF2.1J lines each. *Symbols denote different cell lines.ns = non-significant, ∗p<0.05, ∗∗∗∗p<0.0001.* (See also Figures S4, S5, and S6A).

**Figure 5:**
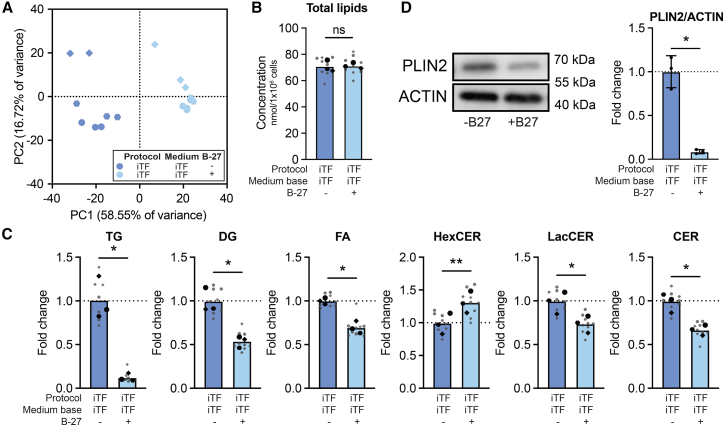
iTF microglia supplemented with B-27 accumulate less LDs (WTC11) (A) PCA plot of unbiased lipidomics. (B) Total lipid concentration in nmol per 1 × 10^6^ cells. Paired *t* test. (C) Lipid class concentration represented as fold change of iTF microglia in iTF medium (-B-27). Paired t-tests with FDR (Benjamini-Hochberg) correction for multiple comparisons. (D) Representative WB and quantification of PLIN2 levels. Paired *t* test. *N* = 3 independent cultures. Data shown as mean ± SEM. (A–C) *N* = 3 independent cultures with 3 technical replicates each. (B and C) *Symbols denote independent cultures. Technical replicates are in gray. The mean of technical replicates is in black*. ns = non-significant, ∗*p* < 0.05, ∗∗*p* < 0.005. (See also Figure S6B).

To identify other factors in the microglial maturation media driving the differences in TG content, we added or removed media components that differed between protocols to the EB microglia maturation medium (Figures 4A, 4B, S5A, and S5B). While factors such as GM-CSF, TGFβ, neurobasal medium (NBM), or dox did not affect TG levels (Figure S5C), the removal of B-27 supplement from EB medium resulted in an increased TG load (Figures 4C and S6A). Strikingly, addition of B-27 supplement to iTF microglia strongly reduced TG levels, as well as its precursors DGs and FAs (Figures 5A–5C and S6B) and PLIN2 protein levels (Figure 5D). As previously observed, HexCERs were oppositely regulated to TGs, with B-27 supplementation increasing HexCERs in iTF microglia (Figure 5C).

### Altered immune states in induced transcription factor microglia driven by B-27 supplement

**Figure 6:**
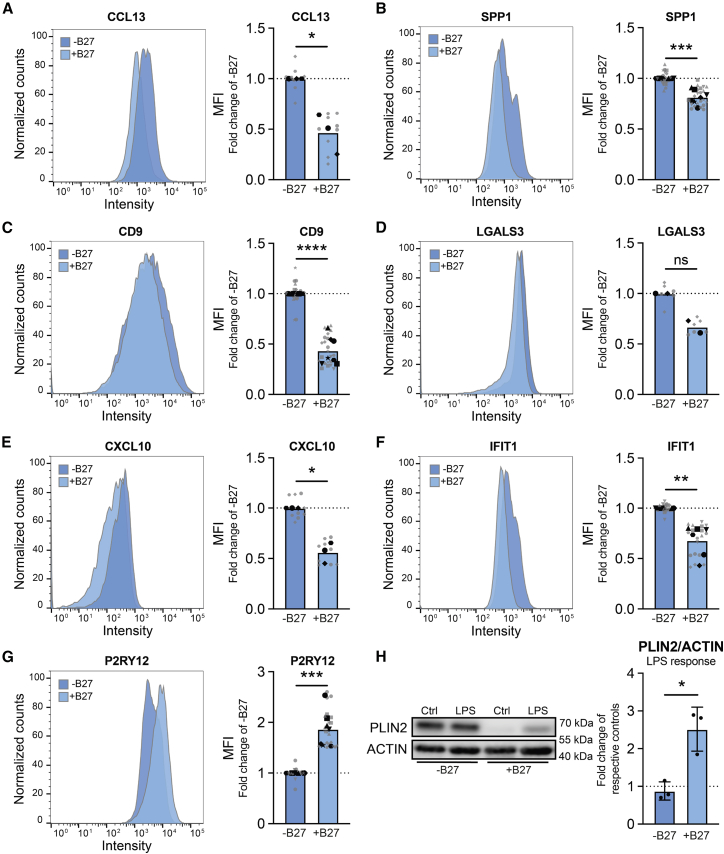
B-27 supplementation alters iTF microglial state (WTC11) (A–G) Representative histogram and quantifications of normalized microglial state markers by flow cytometry. One sample t-tests. N = 2–7 independent cultures with 3 technical replicates each. *Symbols denote independent cultures. Technical replicates are in gray. The mean of technical replicates is in black.* (H) Representative WB and quantification of PLIN2 levels. Paired *t* test. *N* = 3 independent cultures. Data shown as mean ± SEM. ns = non-significant, ∗*p* < 0.05, ∗∗*p* < 0.005, ∗∗∗*p* < 0.0005, ∗∗∗∗*p* < 0.0001.

High TGs have previously been associated with a primed inflammatory phenotype in microglia (Marschallinger et al., 2020). As B-27 lowered TG levels in iTF microglia, we assessed its effect on microglial immune states using flow cytometry (Figures 6A–6G). We observed that B-27 supplementation reduced markers for chemokine-releasing state (CCL13) (Figure 6A), disease-associated markers (SPP1, CD9, and LGALS3) (Figures 6B–6D) and interferon-responsive states (CXCL10 and IFIT1) (Figures 6E and 6F), while increasing the homeostatic marker P2RY12 (Figure 6G). Microglia were previously shown to accumulate LDs after lipopolysaccharide (LPS) stimulation (Marschallinger et al., 2020). Upon the LPS treatment of iTF microglia in their original culture medium, we did not observe a further increase in LDs as assessed by PLIN2 levels. However, when iTF microglia were supplemented with B-27, LPS was able to induce an increase in LDs (Figure 6H), indicating that while B-27 reduces baseline LD levels, it enables microglia to properly adapt their lipidome to immune stimuli.

### L-carnitine partly phenocopies the effect of B-27 on microglial lipidome

**Figure 7:**
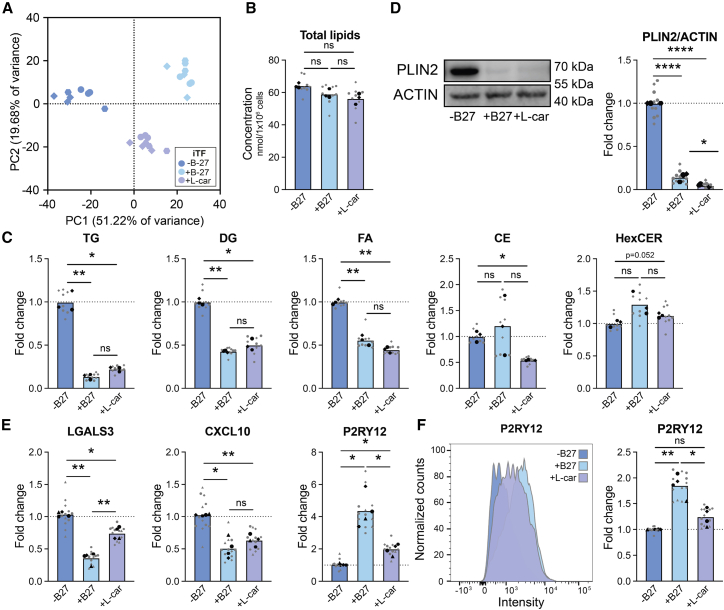
L-carnitine supplementation partly phenocopies B-27 effect on iTF microglial lipidome (WTC11) (A) PCA plot of unbiased lipidomics. (B) Total lipid concentration in nmol per 1 × 10^6^ cells. One-way ANOVA, Tukey’s multiple comparisons post-hoc test. (C) Lipid class concentration represented as fold change of iTF microglia in iTF medium (-B-27). Two-way ANOVA, Tukey’s multiple comparisons post-hoc test. (D) Representative WB and quantification of PLIN2 levels. (A–D) *N* = 3 independent cultures with 3 technical replicates each. (E) mRNA levels of immune state markers represented as fold change of iTF microglia in iTF medium (-B-27). One-way ANOVA, Tukey’s multiple comparisons post-hoc test. (F) Representative histogram and quantifications of normalized microglial homeostatic marker by flow cytometry. One-way ANOVA, Tukey’s multiple comparisons post-hoc test. *The -B-27 and +B-27 data are the same as in*Figures 6B, 6C, 6F, and 6G*.* (E and F) *N* = 4 independent cultures with 3 technical replicates each. *Symbols denote independent cultures. Technical replicates are in gray. The mean of technical replicates is in black.ns = non-significant, ∗p<0.05, ∗∗p<0.005, ∗∗∗∗p<0.0001.* (See also Figure S7).

We noted that the B-27 supplement contains L-car, an essential cofactor for transporting long-chain FAs into mitochondria for fatty acid β-oxidation (FAO) (Longo et al., 2006). Thus, a lack of L-car may lead to FA accumulation in LDs. Indeed, the addition of L-car largely phenocopied the effect of B-27 on the iTF microglial lipidome by reducing TG, DG, and FA content (Figures 7A–7C and S7A). PLIN2 expression was also strongly decreased by L-car (Figure 7D). As with B-27, we evaluated the effects of L-car on microglial state markers (Figures 7E, 7F, S7B, and S7D). L-car significantly reduced *LGALS3* and *CXCL10* mRNA levels (Figure 7E). Like B-27, L-car also increased P2RY12 expression at both the mRNA and protein levels (Figures 7E and 7F), albeit less potently than the full B-27 supplement.

Taken together, these findings suggest that the addition of the B-27 supplement containing L-car in EB microglial maturation medium is responsible for the low TG levels in EB microglia compared to iTF microglia. Lowering TG levels with B-27 or L-car supplementation in iTF microglia correlates with a shift toward a more homeostatic state.

## Discussion

Altered microglial function and lipid metabolism have been implicated in many neurodegenerative diseases, including AD (Tremblay, 2021). A growing body of research uses EB-derived iMGL to generate novel insights on lipid biology in AD etiology and to better understand how AD-associated challenges and variants affect lipid metabolism and microglial function (Dolan et al., 2023; Haney et al., 2024; Podleśny-Drabiniok et al., 2024; Ramaswami et al., 2024; Stephenson et al., 2025; Victor et al., 2022). However, EB-based microglia differentiation protocols are lengthy, costly, and subject to batch-to-batch variability, which restricts their widespread use. As for other cell types (e.g., NGN2 neurons (Zhang et al., 2013), SOX9 and NFIA/NFIB astrocytes (Caiazzo et al., 2015; Canals et al., 2018; Li et al., 2018)), a novel and more scalable protocol to study human microglia *in vitro* has been developed (Dräger et al., 2022). Whether these two methods derive iMGL with similar lipid metabolic states has not been previously addressed. In the present study, through parallel differentiation and comparative lipidomics of iMGL derived from EB and iTF methods, we found that iMGL generated using these approaches differ considerably. iTF microglia contained high levels of TGs and high LD load, while these were lower in EB microglia. Besides the differences in storage lipids, we also noted higher levels of several phospholipid classes and HexCERs in EB microglia compared to their iTF counterparts.

One factor contributing to the lipidomic differences between the iMGL protocols might be microglial maturation states. We found that our EB microglia retained some more macrophage features, and differences in cell identity could affect the lipidome, as shown for other immune cells (Morgan et al., 2024). However, the differences observed are most likely attributable to media composition. Indeed, we found that the B-27 supplement (added to EB but not iTF medium) had major effects on the iMGL lipidome. iMGL cultures require a source of exogenous lipids to support cell growth, which are provided as free FAs in B-27 supplement (only present in EB medium) and/or in Albumax II (0.5× in EB medium, 1× in iTF medium, as a component of the Advanced DMEM/F-12 base). Overall, we found that the lipid composition of EB and iTF media was comparable, indicating that another factor in B-27 underlies the lipidome differences. Importantly, B-27 contains L-car, an essential co-factor for long-chain FA transport from the cytosol to mitochondria via the carnitine palmitoyltransferase 1 (CPT1) transporter (Longo et al., 2006). Addition of L-car phenocopied the effects of B-27 on iMGL lipid composition, reducing levels of FAs, DGs, and TGs and decreasing PLIN2 expression. As B-27, L-car increased the expression of the homeostatic microglia marker P2RY12, uncovering a link between FA metabolism, TG levels, and microglial homeostasis, in line with previous reports (Marschallinger et al., 2020; Stephenson et al., 2025). Importantly, both *in vivo* and *in vitro* (Dräger et al., 2022; Zhang et al., 2016), microglia express minimal L-car synthesizing enzymes such as gamma-butyrobetaine hydroxylase 1 (*BBOX1*), and thus likely rely on exogenous L-car for FAO. Based on our findings, we hypothesize that without exogenously supplied L-car, iMGL cannot oxidize FAs and instead sequester them in LDs as DGs and TGs. Future work should clarify the extent of microglial dependence on L-car and delineate the cellular networks that maintain its supply in the CNS. It is important to note that while B-27 and L-car had similar effects on TGs and LDs, the effect of L-car on P2RY12 was less pronounced, indicating that other bioactive substances present in the B-27 supplement additionally contribute to microglial state and/or function.

Another lipid class regulated by B-27 and L-car was HexCERs. HexCERs are a class of glycosphingolipids consisting of a ceramide backbone linked to either glucose or galactose. Changes in HexCER levels have previously been associated with microglial activation, via direct activation or due to altered metabolism, and may therefore also contribute to changes in microglia downstream of altered (lipid) metabolism (Favret et al., 2024; Shimizu et al., 2023; Wang et al., 2024).

Overall, our findings show that different iMGL protocols lead to vastly different lipidomes, with the recent iTF microglia differentiation method resulting in high TG levels due to the absence of B-27 supplement containing L-car. By the addition of these media components, we describe how iMGL lipid and immune profiles can be drastically altered by culture media ingredients. These findings uncover important considerations in the iPSC modeling of AD and other neurodegenerative diseases in which microglial lipid metabolism has been shown to play a crucial role (Cantuti-Castelvetri et al., 2018; Gabandé-Rodríguez et al., 2019; Safaiyan et al., 2016).

## Methods

### Human induced pluripotent stem cell culture

The WTC11 iTF-iPSC line (male, Asian) was generated as previously described and comprised an inducible CRISPRi machinery in the CLYBL safe-harbor locus, which was left inactive (Dräger et al., 2022). The WTC11 line was provided by Bruce R. Conklin and Li Gan (The J. David Gladstone Institutes), and the WTC11 iTF iPSC line by Martin Kampmann (UCSF). The KOLF2.1J iPSC lines (male, white) (KOLF2.1J Parental and KOLF2.1J CLYBL 6-TF-iMG) were acquired from Jackson Lab.

Frozen iPSCs were thawed, diluted in Advanced DMEM/F-12 (ADF12) (Gibco, 12634028), and centrifuged at 300g for 5 min. Cell pellets were resuspended in Essential 8 Medium (E8) (Gibco, A1517001) supplemented with 0.1% Pen/Strep (P/S) (Fisher Scientific, 11548876) and 5 μM ROCK inhibitor (RI) (Tebu Bio, Y-27632) and plated on Geltrex (Fisher Scientific, A1413302) coated 6-well plates, incubated at 37°C, 5% CO2 with daily refreshments. Cells were passaged twice weekly at ∼75% confluency (1:12-16 split ratio) by dissociation with 1 mM EDTA (Invitrogen, 15575-038) or StemPro Accutase Cell Dissociation Reagent (Accutase) (Gibco, A1110501) at 37°C for 7 min, after which Accutase was diluted 1:5 in ADF12. Cells were centrifuged at 300g for 5 min, and pellets were resuspended in E8 with 5 μM RI. iPSCs were grown to ∼75% confluency and were ≥24h without RI before differentiation. iPSCs were refreshed with Essential 8 Flex Medium (Gibco, A2858591) on weekends. Cultures were monitored daily for sterility and tested monthly for mycoplasma contamination. For cryopreservation, cell pellets were resuspended in KnockOut Serum Replacement (Gibco, 10828028) and 10% dimethyl sulfoxide (Sigma-Aldrich, D2438).

Initial passage numbers for the lines received were P3 for the KOLF2.1J Parental line, P6 for the KOLF2.1J CLYBL 6-TF-iMG line and P18 for the WTC11 iTF iPSC line, where P1 is defined as the passage number when the clone was initially characterized and validated. Cells were expanded for 1–3 passages and stocks were frozen. Identity of the iTF cell lines was confirmed by successful differentiation into iMGL. Quality control of the KOLF2.1J Parental and KOLF2.1J CLYBL 6-TF-iMG lines was performed by JAX Laboratories for karyotype, copy number variation (CNV), confirmation of gene-edited variant sequence (KOLF2.1J CLYBL 6-TF-iMG line), and the absence of viruses, yeast, fungi, and bacteria. Internally, genomic characterization of the KOLF2.1J Parental line was performed by CNV analysis, and deletions were found in *JARID2*, *DTNBP1,* and *ASTN2*, as previously reported (Gracia-Diaz et al., 2024). Pluripotency was assessed by cell providers and not further examined internally.

### Human induced pluripotent stem cell-derived microglia culture and differentiation (embryoid body protocol)

EB microglia were generated as previously described with minor modifications (Feringa et al., 2025; Haenseler et al., 2017; Washer et al., 2022). Briefly, iPSCs were dissociated with Accutase, centrifuged for 5 min at 300g, and cell pellets dissociated to a single-cell suspension in EB induction medium: E8 with 0.1% P/S, 20 ng/mL of recombinant human SCF (Peprotech, 300-07), 50 ng/mL of *E. coli*-derived recombinant human BMP4 (Peprotech, AF-120-05ET), 50 ng/mL of recombinant human VEGF (Peprotech, 100-20), and 5 μM RI for the first 24h. 3 million iPSCs were seeded onto 24-well AggreWell800 plates (STEMCELL Technologies, 17168081) pre-treated with anti-adherence rinsing solution (STEMCELL Technologies, 15973342) in 2 mL of EB induction medium. Cells were cultured in AggreWell at 37°C, 5% CO2 with 75% daily refreshments. On day 7, EBs were harvested and equally distributed to 4 × 10-cm dishes in X-VIVO 15 Serum-free Hematopoietic Cell Medium (Lonza, 02-060Q) with 1% P/S, 1× GlutaMAX (Gibco, 35050038), 0.1 mM 2-mercaptoethanol (BME) (Gibco, 31350010), 25 ng/mL recombinant human IL3 (Peprotech, 200-03) and 100 ng/mL of recombinant human M-CSF (Peprotech, 300-25) and kept at 37°C, 5% CO2 with weekly media refreshments. After 3–4 weeks, EBs started releasing non-adherent macrophage precursors into the medium. Macrophage precursors were harvested by filtering the supernatant during weekly media changes using a 40-μm cell strainer (Greiner, CLY9.1), counted and plated in EB microglia medium (50% ADF12, 50% NBM (Gibco, 21103049) with 0.5% P/S, 1× B-27 Supplement (Gibco, 17504044), 1× GlutaMAX, 0.1 mM BME, 100 ng/mL recombinant human IL-34 (Peprotech, 200-34), 20 ng/mL recombinant human M-CSF and cultivated for 2 weeks with 3 weekly refreshments. The following plating densities were used: 30.000 cells per well for 96-well plates (Ibidi, 89626), 150.000 cells per well for 6-well plates, 1 million cells for 10-cm dishes.

### Media switches for lipidomics (embryoid body protocol)

For media switch experiments, macrophage precursors were plated in one of the following media.

**Table:** 

Base medium	B-27 supplement	GM-CSF, TGFβ	Dox
50% ADF12, 50% NBM	–	–	–
50% ADF12, 50% NBM	–	+	–
50% ADF12, 50% NBM	+	+	–
ADF12	–	+	–
ADF12	+	+	–
ADF12	–	+	+

All media were supplemented with 0.5% P/S, 1× GlutaMAX, 100 ng/mL recombinant human IL-34, and recombinant human M-CSF (20 ng/mL in EB base medium or 50 ng/mL in iTF base medium). Cells were cultivated for 2 weeks with 3 weekly refreshments.

### Human induced pluripotent stem cell-derived microglia culture and differentiation (induced transcription factor protocol)

iTF microglia were generated as previously described with minor modifications (Dräger et al., 2022). Briefly, iPSCs were detached as described above, cell pellets were dissociated to a single-cell suspension in E8 with 0.1% P/S, 5 μM RI, and 2 μg/mL doxycycline (Sigma-Aldrich, D9891), counted and plated on poly-D-lysine (PDL) hydrobromide (Sigma-Aldrich, P6407) and Geltrex double-coated plates/dishes at the following seeding densities: 20.000 cells per well for 96-well plates (Ibidi, 89626), 100.000 cells for 6-well plates, 785.000 cells for 10-cm dishes. On day 2, the medium was replaced with ADF12 with 0.5% P/S, 1× GlutaMAX Supplement, 2 μg/mL dox, 100 ng/mL recombinant human IL34, and 10 ng/mL of recombinant human GM-CSF (Peprotech, 300-03). On day 4, medium was replaced with iTFD4 medium: ADF12 with 0.5% P/S, 1× GlutaMAX Supplement, 2 μg/mL of dox, 100 ng/mL of recombinant human IL34, 10 ng/mL of recombinant human GM-CSF, 50 ng/mL recombinant human M-CSF, and 50 ng/mL of CHO-derived recombinant human TGFβ1 (Peprotech, 100-21C). All experiments were performed on day 8. For experiments including LPS (Invitrogen, 15536286), a full medium refreshment in iTFD4 medium was performed on day 8, and the cells were harvested after 24h. For experiments including B-27 supplement or L-car, the medium was supplemented with 1xB-27 Supplement or 2 μg/mL of L-carnitine hydrochloride (Sigma-Aldrich, C0283) from day 2 onwards, with normal refreshments.

### Lipidomic analysis

#### Sample preparation

iMGL were harvested as follows: cells were washed in DPBS and incubated with Accutase for 7 min before centrifugation at 300*g* for 5 min. Cell pellets were resuspended in ADF12, and 0.5–1.5 million cells were transferred to a conical tube and centrifuged at 300g for 5 min. Cells were resuspended in 1 mL DPBS and transferred into 1.5 mL tubes before centrifugation at 300*g* for 5 min at 4°C. The supernatant was aspirated, and the cell pellet snap-frozen in liquid nitrogen and stored at −80°C until further processing.

#### Sample processing and data analysis

Lipidomic analysis was performed following standardized, quantitative protocols, as previously described (Feringa et al., 2025; Ghorasaini et al., 2021). See also supplemental methods.

#### Lipidomic analysis of embryoid body and induced transcription factor media

To calculate the final concentration of lipids in the media, we performed lipidomics on the media supplements and calculated the final concentration of lipids in the complete medium, accounting for a 1:50 dilution of Albumax II Lipid-Rich BSA (Albumax) (Gibco, 11021029) in the final medium for iTF medium, and a 1:100 dilution of Albumax II and a 1:50 dilution of B-27 supplement in EB medium.

### Quantitative reverse transcription polymerase chain reaction

#### RNA isolation

Cells were lysed in 350 μL of RNA lysis buffer from the ISOLATE II RNA Micro Kit (Meridian Bioscience, BIO-52073) and 10 μL Pierce TCEP-HCl (Thermo Scientific, 20491). RNA extraction was performed according to the manufacturer’s instructions.

#### cDNA conversion

cDNA was synthesized from purified RNA using the SensiFast cDNA Synthesis Kit (Meridian Bioscience, BIO-65054).

#### Quantitative reverse transcription polymerase chain reaction

qRT-PCRs were run on the QuantStudio 5 system (ThermoFisher Scientific) using SensiFast SYBR Lo-ROX Kit (Meridian Bioscience BIO-94020), according to the manufacturer’s instructions. The following primer sets were used.

**Table:** 

Target	Forward	Reverse
*TMEM119*	GGATAGTGGACTTCTTCCGCCA	GGAAGGACGATGGGTAATAGGC
*P2RY12*	TGCCAAACTGGGAACAGGACCA	TGGTGGTCTTCTGGTAGCGATC
*CSF1R*	CACCTTCACCCTCTCTCTGC	AGCATCTTCACAGCCACCTT
*CX3CR1*	CACAAAGGAGCAGGCATGGAAG	CAGGTTCTCTGTAGACACAAGGC
*LYVE1*	GGGTTGGAGATGGATTCGTGG	ATAGGCTGCAAACTGTCGGC
*CXCL10*	GTGGCATTCAAGGAGTACCTC	TGATGGCCTTCGATTCTGGATT
*IFIT1*	GCCTTGCTGAAGTGTGGAGGAA	ATCCAGGCGATAGGCAGAGATC
*STAT1*	ATGGCAGTCTGGCGGCTGAATT	CCAAACCAGGCTGGCACAATTG
*STAT2*	CAGGTCACAGAGTTGCTACAGC	CGGTGAACTTGCTGCCAGTCTT
*IRF7*	CCACGCTATACCATCTACCTGG	GCTGCTATCCAGGGAAGACACA
*LGALS3*	CCATCTTCTGGACAGCCAAGTG	TATCAGCATGCGAGGCACCACT
*ACTB*	CACCATTGGCAATGAGCGGTTC	AGGTCTTTGCGGATGTCCACGT

### Immunocytochemistry and imaging

iMGL were fixed with 4% paraformaldehyde (Sigma-Aldrich, P6148) for 25 min at room temperature (RT) before permeabilization with 0.1% Triton X-100 (Fisher Chemical, 10254640) for 5 min at RT and 45 min blocking in PBS with 0.1% Triton X-100, 5% normal goat serum (Gibco, 11540526) and 2% BSA (Roche, 10735086001). Cells were incubated with the following primary antibodies for 2h at RT: anti-perilipin 2 (Proteintech, 15294-1-AP) and anti-Iba1 (FUJIFILM Wako Pure Chemical Corporation, 019–19741). Then, cells were washed 3× in PBS with 0.1% Triton X-100 before incubation with Alexa-Fluor secondary antibodies (Invitrogen, 1:1000), DAPI (Carl Roth, 6843.1), and phalloidin iFluor 647 Reagent (Abcam, ab176759), where applicable. Finally, cells were washed 3× with PBS and imaged on the CellInsight CX7 LED Pro HCS Platform (Fisher Scientific, Hampton, NH, USA) or the Nikon Ti-Eclipse microscope, equipped with a confocal scanner model A1R+, using a 40× oil immersion objective (NA = 1.3). Image analysis was performed using Columbus version 2.5.2 (PerkinElmer, Waltham, MA, USA) after imaging on CX7, or using Fiji (Schindelin et al., 2012) for confocal imaging on the Nikon Ti-Eclipse.

### Western blot

Cells were lysed with LSB and samples denatured for 5 min at 95°C and shortly centrifuged before loading onto a 4–15% Criterion TGX Stain-free gel (BIO-RAD, 5678085). Gels were run at 90V for 30 min, followed by 45 min at 150V before transfer using the Trans-Blot Turbo RTA Midi 0.45 μm LF PVDF Transfer kit (BIO-RAD, 1704275) and the *Trans*-blot Turbo Transfer System (BIO-RAD, 1704150). Membranes were blocked 1h at RT in 5% BSA (Sigma, 10735086001) before incubation with antibodies against PLIN2 (Proteintech, 15294-1-AP, 1:5000), GAPDH (elabscience, E-AB40337, 1:3000), or ACTIN (Sigma-Aldrich, MAB1501, 1:5000) at 4°C on a rocking plate overnight. Then, membranes were incubated 1h on a shaker at RT with secondary antibodies polyclonal HRP (Agilent, P044801, 1:5000) and IRDye 800CW (LI-COR, 926–32210, 1:10000) or IRDye 680RD (LI-COR, 926–68071, 1:10000). Membranes were scanned using the LI-COR Odyssey Fc Imaging System (LI-COR, Cambridge, UK). Analysis was performed using Image Studio Lite 5.2.5 Software (LI-COR, Cambridge, UK) by calculating the median intensity of bands and subtracting the background signal above and below bands.

### Flow cytometry

On day 8, iTF microglia were detached with TrypLE Express (Gibco, 12605-028) for 10 min at 37°C, washed with ADF12 and centrifuged at 300g for 5 min. For extracellular markers, cells were incubated with antibodies against CD9 (BioLegend, 312104, 1:200), P2RY12 (BioLegend, 392108, 1:50), or LGALS3 (BioLegend, 125410, 1:50) and FC block (BioLegend, 422302, 1:200) in FACS buffer containing 3% BSA and 0.5 mM EDTA, for 30 min at 4°C. For intracellular markers, cells were fixed for 20 min at RT using the eBioscience Intracellular Fixation and Permeabilization Buffer Set (Invitrogen, 88-8824-00), before adding antibodies against IFIT1 (Cell Signaling, 20329S, 1:100), CXCL10 (BioLegend 519504, 1:100), SPP1 (eBioscience, 50-9096-42, 1:50) or CCL13 (R&D systems, IC327G, 1:50) for 30 min at RT. Samples were analyzed on a BD LSR Fortessa X14 using BD FACSDiva software. Mean fluorescence intensity was calculated using FlowJo after gating for live, single cells.

### Statistical analyses

Statistical analyses were performed in GraphPad Prism version 10.4.2 (GraphPad Software, Boston, MA, USA). Paired t-tests (where *p*-values were corrected for multiple comparisons where applicable, using the Benjamini-Hochberg False Discovery Rate (FDR) method), unpaired t-tests (where *p*-values were corrected for multiple comparisons where applicable, using the Benjamini-Hochberg FDR method), one-sample t-tests, one- and two-way ANOVA (with Tukey’s multiple comparisons post-hoc test) were used as indicated. Adjusted *p*-values are reported where applicable. Statistical tests and sample sizes are indicated in figure legends. All statistical testing was performed on the mean of technical replicates for each independent experiment where applicable.

## Resource availability

### Lead contact

Further inquiries and resource requests should be directed to Dr. Rik van der Kant (r.h.n.vander.kant@vu.nl).

### Materials availability

The KOLF2.1J parental (JIPSC001000) and KOLF2.1J CLYBL 6-TF-iMG (JIPSC002072) iPSC lines are available from the JAX repository. The WTC11 iTF iPSC line is available through Coriell (AICS-0090-391).

### Data and code availability

All lipidomic datasets will be published on the www.neurolipidatlas.com repository and deposited in the Metabolomics Workbench National Metabolomics Data Repository: PR002792 (https://doi.org/10.21228/M8RV8C) as of the date of publication (Sud et al., 2016). Raw data files are also provided as Data S1, S2, S3, S4, S5, S6, S7, S8, S9, and S10.

## Acknowledgments

This work was supported by an 10.13039/100000957Alzheimer's Association Grant through the AD Strategic Fund (ADSF-21-831212-C) to RvdK and MK, a grant from the 10.13039/100007625Cure Alzheimer's Fund to RvdK, an Alzheimer’s Association Zenith award (ZEN-22-969903) and 10.13039/100014989Chan Zuckerberg Initiative award (CP2-1-0000000332) to MK, an 10.13039/100000957Alzheimer's Association grant (AARF-22-973222) and a grant from the 10.13039/100001167Larry L. Hillblom Foundation (2022-A-016-FEL) to AM. LE was supported by the 10.13039/501100004350Studienstiftung des Deutschen Volkes and the 10.13039/100018173International Max Planck Research School for the Mechanisms of Mental Function and Dysfunction.

We thank members of the van der Kant lab, Ruud Wijdeven, Kim de Kleijn, and Matthijs Verhage for their invaluable feedback on this work. We thank Niek Blomberg for performing the lipidomic measurements, Lian Wang for offering support during data submission, Bill Skarnes and Michael Ward for providing early access to the KOLF2.1J CLYBL 6-TF-iMG iPSC line, and Bruce R. Conklin and Li Gan for generously sharing cell lines.

## Author contributions

ATR designed, performed, and analyzed experiments on all aspects of the study and wrote the article; AM performed and analyzed flow cytometry experiments; SJK assisted with flow cytometry experiments and provided bioinformatics support; LE optimized the method for EB microglial differentiation; DKV supervised method optimization for EB microglial differentiation; MK supervised flow cytometry experiments; MG supervised lipidomic measurements; RvdK designed and supervised experiments on all aspects of the study and wrote the article.

## Declaration of interests

MK is a co-scientific founder of Montara Therapeutics and serves on the Scientific Advisory Boards of Engine Biosciences, Casma Therapeutics, Alector, Montara Therapeutics, and Theseus, and is an advisor to Modulo Bio and Recursion Therapeutics.

The other authors declare no competing interests.

## Supplemental information

Document S1. Figures S1–S7 and supplemental methods

Data S1. Lipidomic data file of EB and iTF microglia (WTC11), related to Figure 2

Data S2. Lipidomic data file of EB and iTF microglia with medium switch (WTC11) (dataset 1), related to Figure 3

Data S3. Lipidomic data file of EB and iTF microglia with medium switch (WTC11) (dataset 2), related to Figure 3

Data S4. Lipidomic data file of EB microglia grown with and without B-27 (WTC11), related to Figure 4

Data S5. Lipidomic data file of EB microglia grown with and without B-27 (KOLF2.1J) vs. iTF microglia, related to Figure 4

Data S6. Lipidomic data file of iTF microglia grown without and with B-27 (WTC11), related to Figure 5

Data S7. Lipidomic data file of EB and iTF microglia (KOLF2.1J), related to Figure S2

Data S8. Lipidomic data file and calculations of EB and iTF media lipid compositions, related to Figure S4

Data S9. Lipidomic data file of EB microglia grown with and without GM-CSF, TGFβ, NBM, dox (KOLF2.1J), related to Figure S5

Data S10. Lipidomic data file of iTF microglia supplemented with B-27 or L-carnitine (WTC11), related to Figure 7

Document S2. Article plus supplemental information
