# Supplementary material for: Comparative lipidomics of iPSC-derived microglia protocols reveal lipid droplet and immune differences mediated by media composition
Source: Stem Cell Reports. 2026 Jan 8;21(2):102779. doi: 10.1016/j.stemcr.2025.102779 (PMC12903089; doi:10.1016/j.stemcr.2025.102779)
Supplement: Document S1. Figures S1–S7 and supplemental methods [file mmc1.pdf]

Stem Cell Reports, Volume *21*

Supplemental Information

Comparative lipidomics of iPSC-derived microglia protocols reveal lipid droplet and immune differences mediated by media composition

Aiko Toda Robert, Amanda McQuade, Sascha J. Koppes-den Hertog, Lena Erlebach, Deborah Kronenberg-Versteeg, Martin Kampmann, Martin Giera, and Rik van der Kant

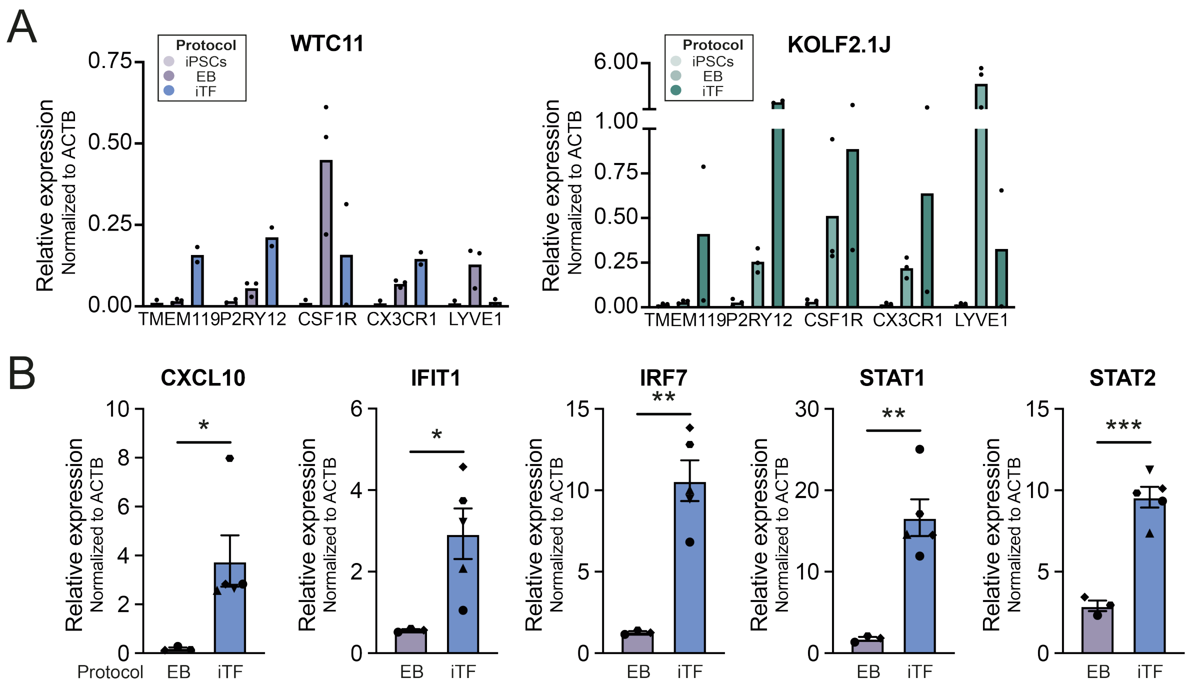

Supplemental figure S1. mRNA expression of microglia/macrophage signature genes in iPSC-derived EB and iTF microglia. A) mRNA expression of microglia marker genes in iPSCs, EB microglia and iTF microglia in WTC11 line (left) and KOLF2.1J line (right). N=3 independent cultures for EB microglia, N=2 independent cultures for iTF microglia. Data represented as relative expression (2^(-ΔCt)) values multiplied by 100 for ease of visualization. B) mRNA levels of interferon-responsive microglia markers and interferon regulatory genes in EB vs iTF microglia (WTC11). N=3 independent cultures for EB microglia, N=5 independent cultures for iTF microglia. Data represented as relative expression (2^(-ΔCt)) values multiplied by 1000 for ease of visualization. (Related to Figure 1)

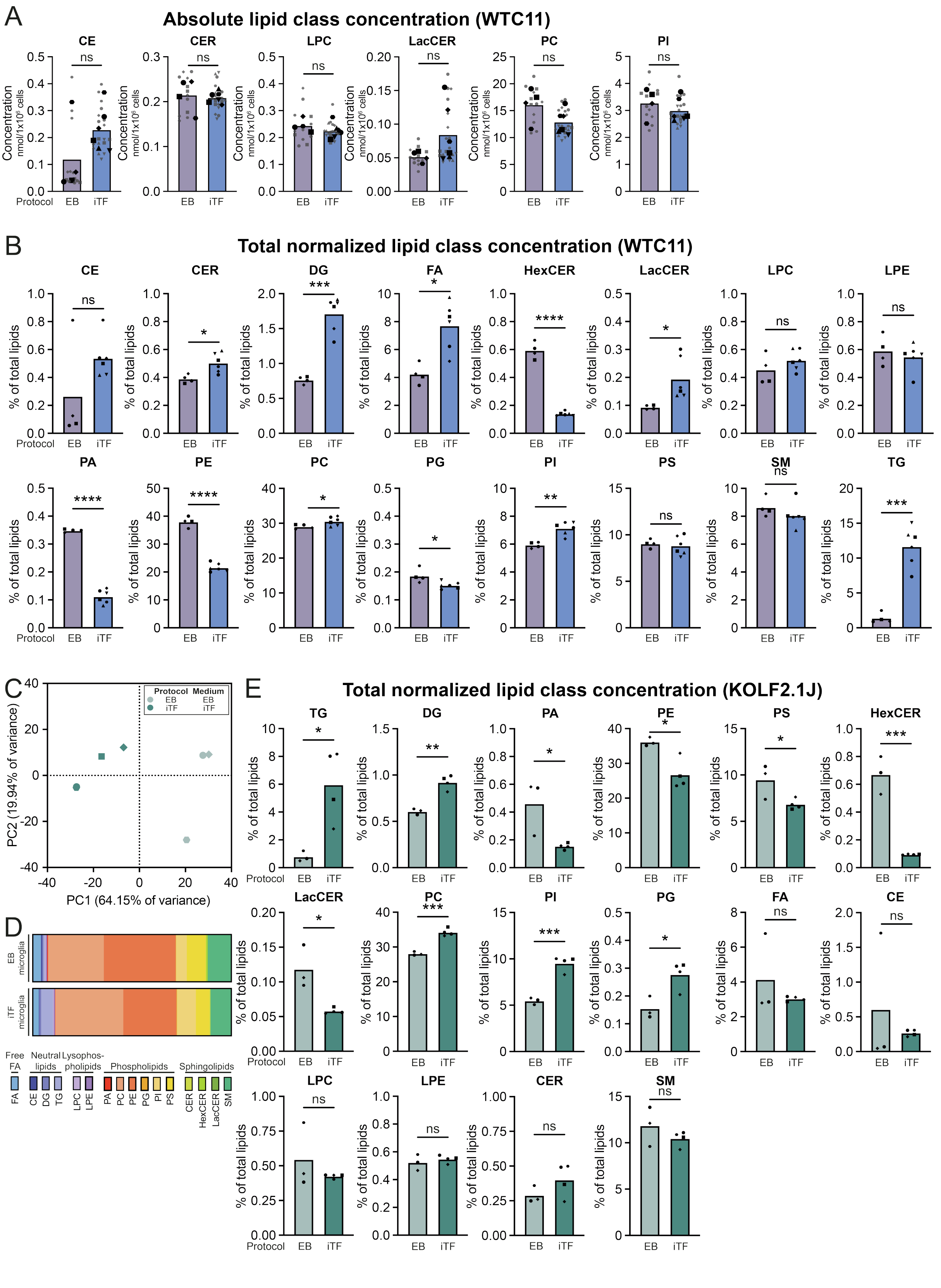
A
Absolute lipid class concentration (WTC11)
CE
CER
LPC
LacCER
PC
PI
ns
Concentration nmol/1x10⁶ cells
Protocol
EB
iTF
B
Total normalized lipid class concentration (WTC11)
CE
CER
DG
FA
HexCER
LacCER
LPC
LPE
PA
PE
PC
PG
PI
PS
SM
TG
% of total lipids
Protocol
EB
iTF
C
Protocol Medium
EB EB
iTF iTF
PC2 (19.94% of variance)
PC1 (64.15% of variance)
D
EB microglia
iTF microglia
Free FA
Neutral lipids
Lysophospholipids
Phospholipids
Sphingolipids
FA
CE
DG
TG
LPC
LPE
PA
PC
PE
PG
PI
PS
CER
HexCER
LacCER
SM
E
Total normalized lipid class concentration (KOLF2.1J)
TG
DG
PA
PE
PS
HexCER
LacCER
PC
PI
PG
FA
CE
LPC
LPE
CER
SM
% of total lipids
Protocol
EB
iTF

Supplemental figure S2. Lipidomic analysis of iPSC-derived EB and iTF microglia (WTC11 and KOLF2.1J). A) Lipid class concentration in nmol per 1 million cells (WTC11). Non-significantly different classes. Unpaired t- tests with FDR (Benjamini-Hochberg) correction for multiple comparisons (performed on all lipid classes in Figure 2D and S2A). *Symbols denote independent cultures. Technical replicates are in grey. The mean of technical replicates is in black.* B) Lipid class concentration per 1 million cells represented as percentage of total lipid fraction (WTC11). Unpaired t-tests with FDR (Benjamini-Hochberg) correction for multiple comparisons. A-B) N=4 and N=6 independent cultures for EB and iTF microglia, respectively with 3 technical replicates for each independent culture. C) PCA plot of unbiased lipidomic analysis (KOLF2.1J). D) Average distribution of lipid classes as percentage of total lipids (KOLF2.1J). E) Lipid class concentration per 1 million cells represented as percentage of total lipid fraction (KOLF2.1J). Unpaired t-tests with FDR (Benjamini-Hochberg) correction for multiple comparisons. C-E) N=3 and N=4 independent cultures for EB and iTF microglia, respectively. *Symbols denote independent cultures.* (Related to Figure 2)

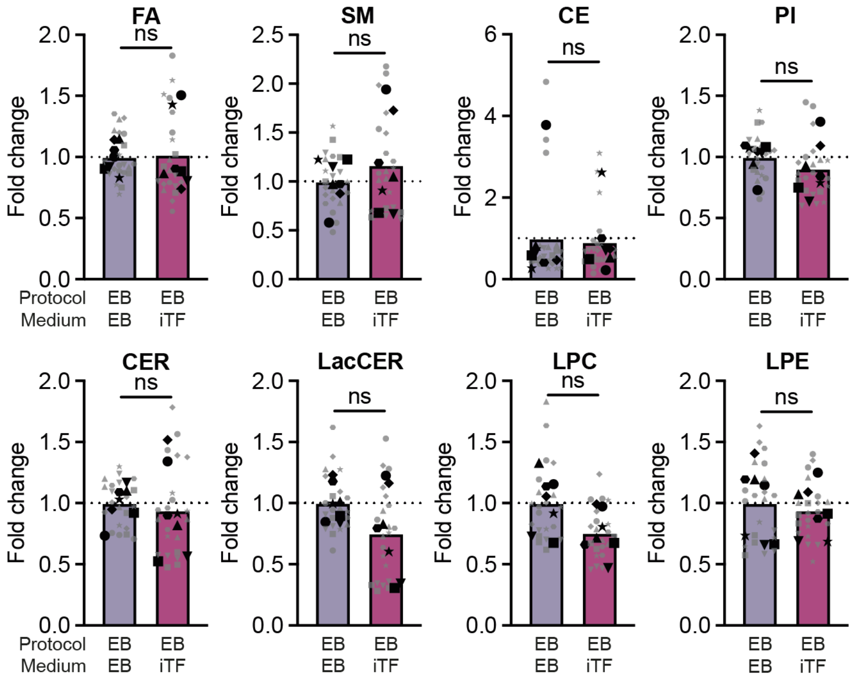

Supplemental figure S3. Lipidomic comparison of iPSC-derived EB microglia grown in EB and iTF medium (WTC11). Lipid class concentration represented as fold change of EB microglia in EB medium sample mean. Non-significantly different classes. Paired t-tests with FDR (Benjamini-Hochberg) correction for multiple comparisons (performed on all lipid classes in Figure 3C and S3). N=7 independent cultures with 3 technical replicates each. *Symbols denote independent cultures. Technical replicates are in grey. The mean of technical replicates is in black.* (Related to Figure 3)

A

	EB microglia	iTF microglia
**Medium base**	0.5x Advanced DMEM/F-12<br>0.5x Neurobasal medium	1x Advanced DMEM/F-12
**Lipid sources**<br>*in medium base*<br>*supplement*	 0.5x AlbuMAX II<br>B-27 supplement	 1x AlbuMAX II

B

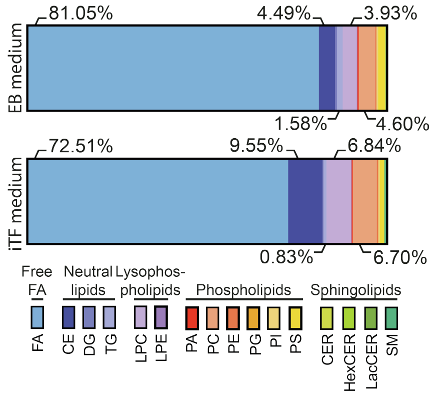

C

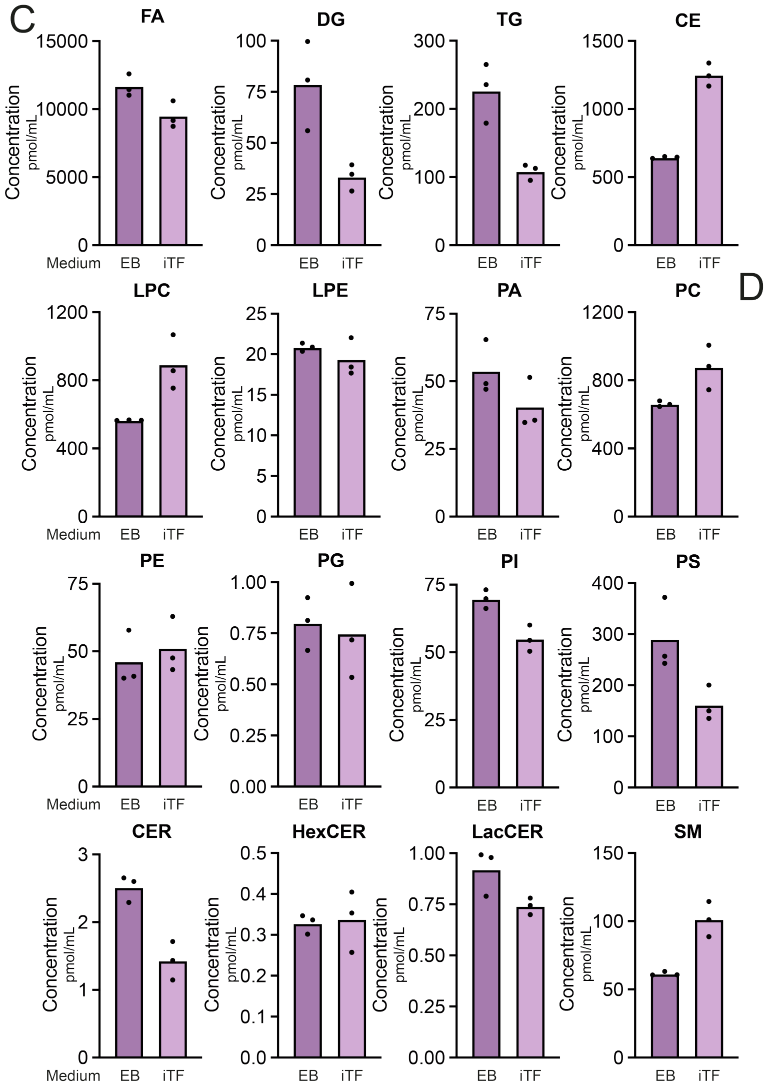

D

B-27 supplement components	iTF medium
**Vitamins**	
Biotin	✓
DL Alpha tocopherol acetate	x
DL Alpha tocopherol	x
Vitamin A (acetate)	x
**Proteins**	
BSA, fatty acid free Fraction V	✓
Catalase	x
Human recombinant insulin	✓
Superoxide dismutase	x
**Other**	
Corticosterone	x
D-Galactose	x
Ethanolamine HCl	✓
Glutathione (reduced)	✓
L-Carnitine HCl	x
Linoleic Acid	✓
Linolenic Acid	✓
Progesterone	x
Putrescine 2HCl	✓
Sodium Selenite	✓
T3 (triodi-l-thyronine)	x

Supplemental figure S4. Lipidomic comparison of EB and iTF media composition. A) Overview of the different lipid sources in microglia maturation media. B) Average distribution of lipid classes as percentage of total lipids. C) Lipid class concentration in pmol/mL. N=3 technical replicates. D) Overview of B-27 supplement composition and their presence or absence in iTF medium. (Related to Figure 4)

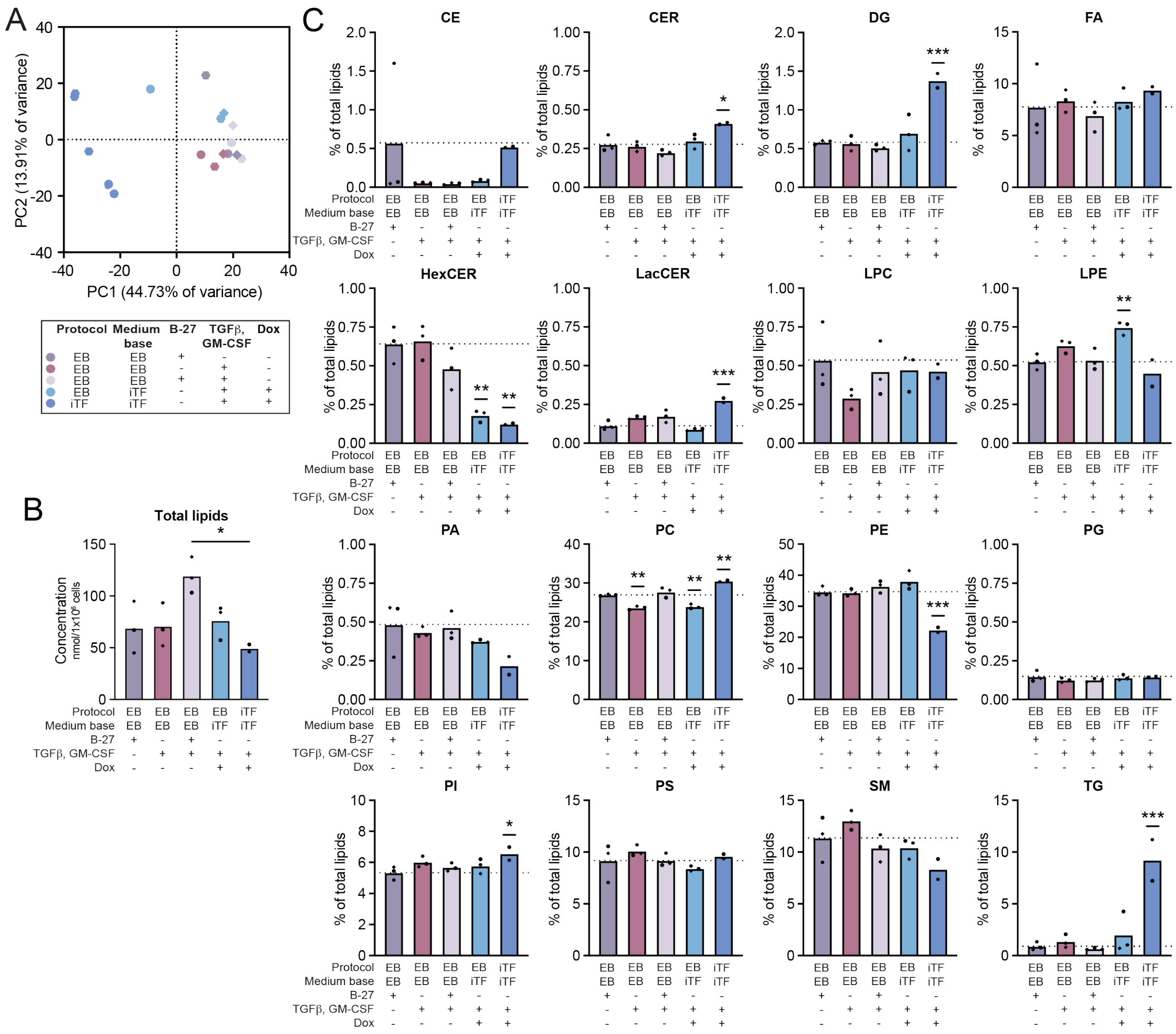

Supplemental figure S5. Lipidomic comparison of EB microglia grown in the presence or absence of TGFb, GM-CSF, NBM and dox (KOLF2.1J). A) PCA plot of unbiased lipidomic analysis. B) Total lipid concentration in nmol per 1 million cells. One-way ANOVA, Tukey's multiple comparisons post-hoc test. C) Lipid class levels shown as percentage of total lipid fraction. One-way ANOVA, Tukey's multiple comparisons post-hoc test. A-C) N=3 independent cultures for EB microglia and N=2 for iTF microglia, taken as the mean of 3 technical replicates. *Symbols denote independent cultures.* (Related to Figure 4)

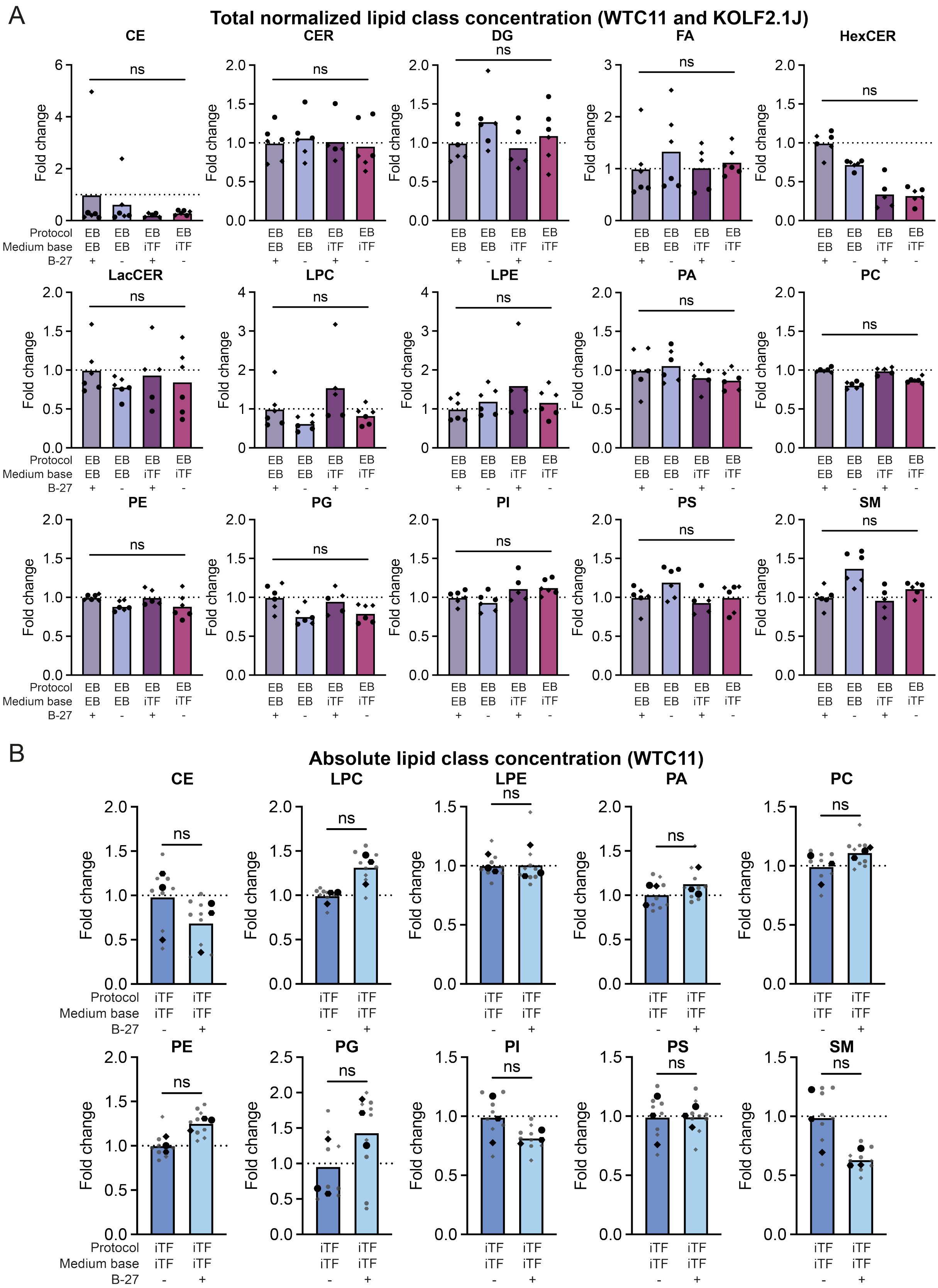
A
Total normalized lipid class concentration (WTC11 and KOLF2.1J)
CE
CER
DG
FA
HexCER
LacCER
LPC
LPE
PA
PC
PE
PG
PI
PS
SM
ns
Fold change
Protocol
Medium base
B-27
EB
iTF
+
-
B
Absolute lipid class concentration (WTC11)
CE
LPC
LPE
PA
PC
PE
PG
PI
PS
SM
ns
Fold change
Protocol
Medium base
B-27
iTF
-
+

Supplemental figure S6. Lipidomic comparison of iMGL grown in the presence or absence of B-27 supplement (EB and iTF microglia). A) Lipid class concentration represented as fold change of EB microglia in EB medium sample mean (WTC11 & KOLF2.1J). Non-significantly different lipid classes. Two-way ANOVA, Tukey's multiple comparisons post-hoc test (performed on all lipid classes in Figure 4C and S6A). N=3 independent cultures from WTC11 and KOLF2.1J lines each. *Symbols denote different cell lines.* (Related to Figure 4). B) Lipid class concentration represented as fold change of iTF microglia in iTF medium (- B-27) sample mean (WTC11). Non-significantly different lipid classes. Paired t-tests with FDR (Benjamini-Hochberg) correction for multiple comparisons (performed on all lipid classes in Figure 5C and S6B). N=3 independent cultures, with 3 technical replicates per condition. *Symbols denote independent cultures. Technical replicates are in grey. The mean of technical replicates is in black.* (Related to Figure 5)

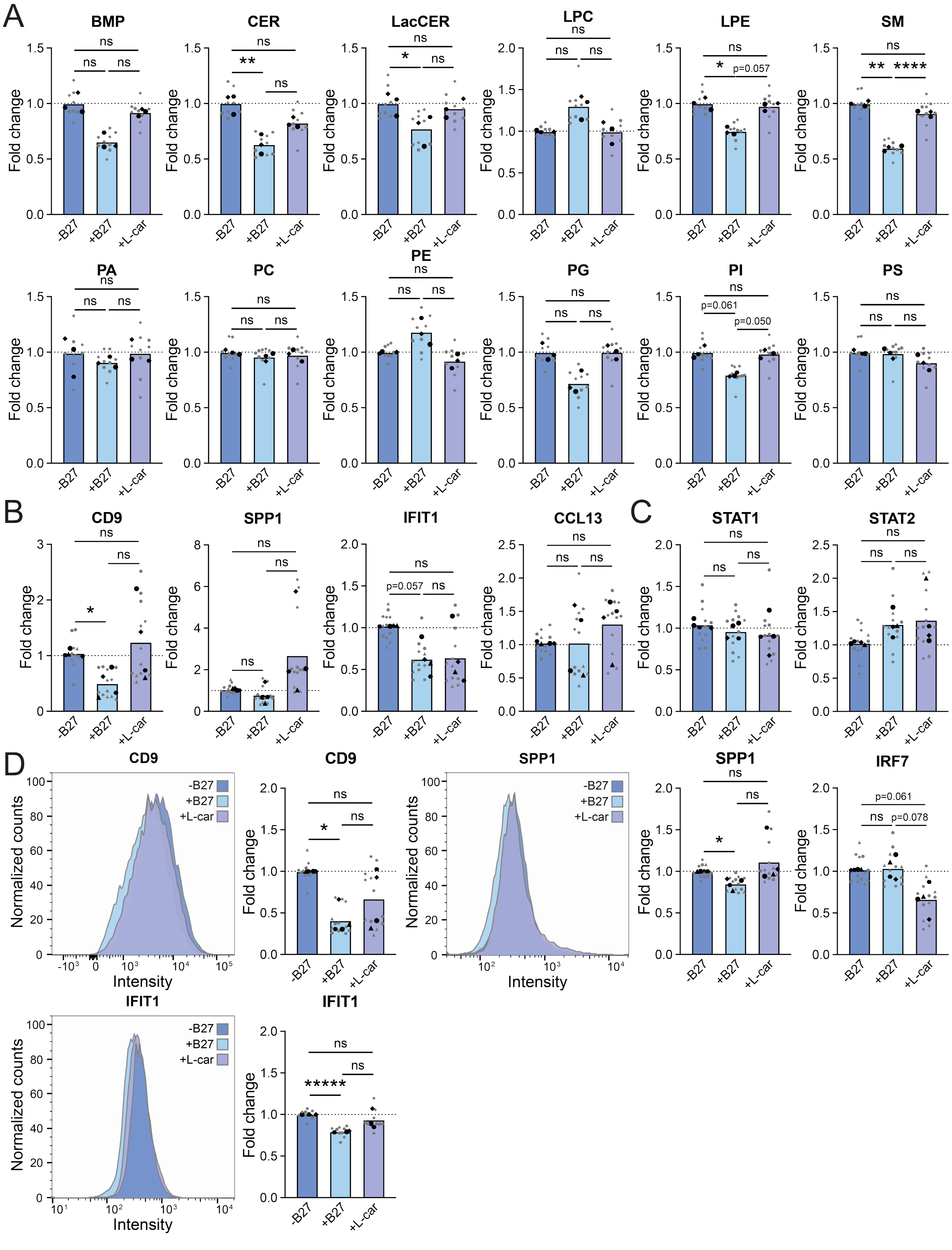
A
BMP
CER
LacCER
LPC
LPE
SM
PA
PC
PE
PG
PI
PS
B
CD9
SPP1
IFIT1
CCL13
C
STAT1
STAT2
D
CD9
SPP1
IRF7
IFIT1
Fold change
Normalized counts
Intensity
-B27
+B27
+L-car
ns
p=0.057
p=0.061
p=0.050
p=0.078

Supplemental figure S7. Effect of L-carnitine supplementation on iTF microglial lipidome and immune markers. A) Lipid class concentration represented as fold change of iTF microglia in iTF medium (- B-27) sample mean (WTC11). Non-significantly different lipid classes between -B27 and +L-carnitine (L-car) condition. Two-way ANOVA, Tukey's multiple comparisons post-hoc test (performed on all lipid classes in Figure 7C and S7A). N=3 independent cultures, with 3 technical replicates per condition. B) mRNA levels of immune markers with B-27 or L-carnitine supplementation. C) mRNA levels of interferon-responsive microglia markers with B-27 or L-carnitine supplementation. D) Representative histogram and quantifications of normalized microglial state markers determined by flow cytometry. One-way ANOVA, Tukey's multiple comparisons post-hoc test. B-D) N=4 independent cultures, with 3 technical replicates each. *Symbols denote independent cultures. Technical replicates are in grey. The mean of technical replicates is in black.* (Related to Figure 7)

Supplemental methods

Lipidomic analysis

Sample processing and data analysis

Briefly, 25 μL of the Lipidyzer internal standard mix containing 54 deuterated standards, was added to the cell pellet. Extraction was performed using a methyl tert-butyl ether-based method. After drying under a gentle stream of nitrogen, samples were dissolved in running buffer consisting of methanol:dichloromethane (1:1) containing 10 mM ammonium acetate, before injection into the Lipidyzer platform consisting of a SCIEX QTRAP 5500 mass spectrometer equipped with an SelexION DMS interface and a Nexera X3 UHPLCsystem. SLA software was used to process data files and report lipid class, species concentration and composition values (Su et al., 2021). Lipidyzer data analysis was carried out on SODA-light, a built-in data browser for the Neurolipid Atlas repository. Lipid species concentration datasets were filtered to retain species with values at least twice the blank in ≥80% of samples. Species below this threshold were excluded, except those uniquely detected in one group and present in ≥60% of its samples. SODA-light version 0.2 was used to generate the lipidomic data files in this manuscript. Lipidomic data are reported as absolute concentration (nmol/1x10^6 cells for cells, pmol/mL for media) and/or as a fraction of total lipids when the total lipid concentration between samples differed considerably. Lipidomic samples measured in separate runs were corrected for batch effect based on quality control samples included in each run, when all sample groups were present in all datasets. Where batch correction was not possible, data are reported as a fraction of total lipids. SODA-light is a development branch of iSODA [https://github.com/ndcn/soda-ndcn] and part of the Neurolipid Atlas. FAIR principles were followed for lipidomic data formatting and storage.

References

Su, B., Bettcher, L.F., Hsieh, W.Y., Hornburg, D., Pearson, M.J., Blomberg, N., Giera, M., Snyder, M.P., Raftery, D., Bensinger, S.J., et al. (2021). A DMS Shotgun Lipidomics Workflow Application to Facilitate High-Throughput, Comprehensive Lipidomics. J Am Soc Mass Spectrom *32*, 2655–2663. https://doi.org/10.1021/jasms.1c00203.
